# FunTuple: A New N-tuple Component for Offline Data Processing at the LHCb Experiment

**DOI:** 10.1007/s41781-024-00116-1

**Published:** 2024-02-24

**Authors:** Abhijit Mathad, Martina Ferrillo, Sacha Barré, Patrick Koppenburg, Patrick Owen, Gerhard Raven, Eduardo Rodrigues, Nicola Serra

**Affiliations:** 1 University of Zürich, Zürich, Switzerland; 2 European Organization for Nuclear Research (CERN), Geneva, Switzerland; 3 The University of Manchester, Manchester, UK; 4 Nikhef National Institute for Subatomic Physics, Amsterdam, Netherlands; 5 VU University Amsterdam, Amsterdam, Netherlands; 6 Oliver Lodge Laboratory, University of Liverpool, Liverpool, UK

**Keywords:** High-energy-physics, LHCb experiment, Data processing and offline analysis

## Abstract

The offline software framework of the LHCb experiment has undergone a significant overhaul to tackle the data processing challenges that will arise in the upcoming Run 3 and Run 4 of the Large Hadron Collider. This paper introduces FunTuple, a novel component developed for offline data processing within the LHCb experiment. This component enables the computation and storage of a diverse range of observables for both reconstructed and simulated events by leveraging on the tools initially developed for the trigger system. This feature is crucial for ensuring consistency between trigger-computed and offline-analysed observables. The component and its tool suite offer users flexibility to customise stored observables, and its reliability is validated through a full-coverage set of rigorous unit tests. This paper comprehensively explores FunTuple’s design, interface, interaction with other algorithms, and its role in facilitating offline data processing for the LHCb experiment for the next decade and beyond.

## Introduction

The LHCb experiment, located at Point 8 of the Large Hadron Collider (LHC) [1] at CERN, is a forward-arm spectrometer designed to study the decays of beauty and charm hadrons [2, 3]. In the initial two runs of the LHC, during 2010–2018, the experiment (mainly) collected proton–proton collision data corresponding to a total integrated luminosity of 9$\,\text {fb} ^{-1}$. As preparations intensify for Run 3,^1^ where the LHC’s instantaneous luminosity is anticipated to surge by a factor of 5 compared to the preceding runs, the LHCb experiment is poised to enhance its capabilities even further. The upgraded detector [4] and data acquisition system will allow for improved vertexing and trigger efficiency [5]. This enhancement facilitates the exploration of exceedingly rare decays [6] while also facilitating the probing of deviations from Standard Model predictions with unparalleled precision [7–9].

The advent of Run 3 data acquisition presents significant hurdles for the LHCb data processing framework. Notably, the data volume from LHCb ’s Run 3 is projected to surge by over 15 times compared to prior runs [10]. Consequently, management of petabytes of processed data and effectively incorporating distributed computing resources present significant challenges [11, 12]. In light of these challenges, a comprehensive redesign of both the trigger and offline data processing pipelines is imperative [10, 11]. This paper concentrates on the offline data processing pipeline, specifically highlighting the development of a new component called FunTuple [13] facilitating analysis of Run 3 data and beyond.

In the initial LHC runs, LHCb ’s trigger and offline reconstruction applications, Moore [14] and Brunel [15], operated independently from the DaVinci application [16] employed for offline data processing. Besides executing offline event selection, the DaVinci application was used to process and store data for subsequent analysis. This task was accomplished via the DecayTreeTuple algorithm [17],^2^ which recorded a specific set of observables into output files. First, due to the segregation of trigger and offline frameworks, the equivalence between trigger-computed observables and those analysed offline was not guaranteed. Second, users lacked the flexibility to customise the set of observables recorded, which is essential in light of the anticipated data volume surge for Run 3 and Run 4. Furthermore, as part of its strategy to tackle the forthcoming data processing challenges in Run 4 and beyond, the LHCb experiment plans to implement a new event model based on *Structure of Arrays* (SoA), which will facilitate vectorised processing of data [20]. Substantial enhancements were also made to the trigger reconstruction algorithms that facilitated retirement of the Brunel package, which was responsible for offline reconstruction [21–24]. Consequently, the development of new offline algorithms becomes imperative to accommodate these changes.

**Fig. 1:**
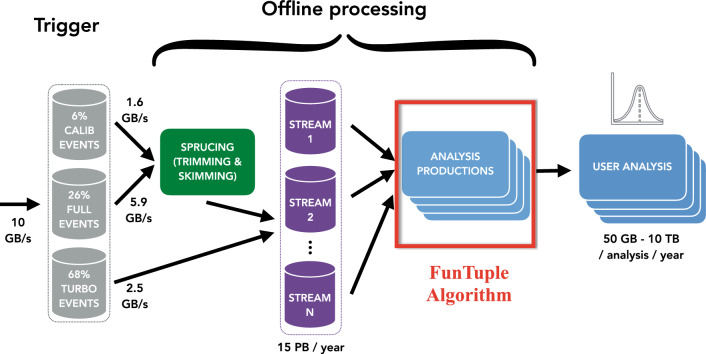
Data flow diagram for Run 3 data processing showing the placement of the FunTuple component. Figure adapted from Ref. [30]

To overcome these hurdles, a strategic choice was made to leverage tools developed for the trigger system within the offline software framework. This led to the development of a new component, FunTuple [13], short for *Fun*ctional n*Tuple*, which is tailored for processing Run 3 and Run 4 data. The FunTuple component introduces enhancements to the previous workflow. First, it guarantees the consistency between trigger-computed observables and those subjected to offline analysis. Second, FunTuple, along with all its dependencies, is entirely templated in C++, allowing it to support both legacy and upcoming event models planned for future LHC runs. The templated design along with the SoA event model enables the component to leverage *Single Instruction Multiple Data* (SIMD) vectorisation. Last, it offers users the flexibility to efficiently tailor the list of recorded observables, an important feature given the expected surge in data volume for Run 3 and Run 4. This component is configured with a robust suite of tools designed for the second stage of the LHCb trigger system, known as Throughput Oriented (ThOr) functors [25–27]. These functors are designed to deliver high-speed in the trigger’s demanding throughput environment and are adept at computing topological and kinematic observables. FunTuple utilises these functors to compute a diverse range of observables and writes a TTree in the ROOT N-tuple format.^3^ The N-tuple format is widely used in the High Energy Physics community to store flattened data in a tabular format [29]. Furthermore, the component’s lightweight design ensures simplified maintenance and seamless knowledge transfer. As depicted in Fig. 1, the FunTuple component plays a central role, bridging the gap between the offline data processing stage (Sprucing) and the subsequent user analysis stages [30]. In the Sprucing stage, the data are slimmed and skimmed before being saved to disk as part of the offline data processing workflow. The placement of FunTuple underscores its critical role in LHCb ’s analysis productions [31], facilitating the storage of experiment-acquired data in a format suitable for subsequent offline analysis.

## Design and Interface

FunTuple is a novel component integral to the LHCb experiment’s data processing infrastructure. It is a c++ [32] class built upon the Gaudi functional framework [33], and it offers a user-friendly Python [34] interface. The flexibility of the FunTuple component lies in its templated design, allowing it to accommodate various types of input data. As a result, for Run 3, it is available in the three distinct flavours FunTuple_Particles, FunTuple_MCParticles and FunTuple_Event hereafter described.

**Fig. 2:**
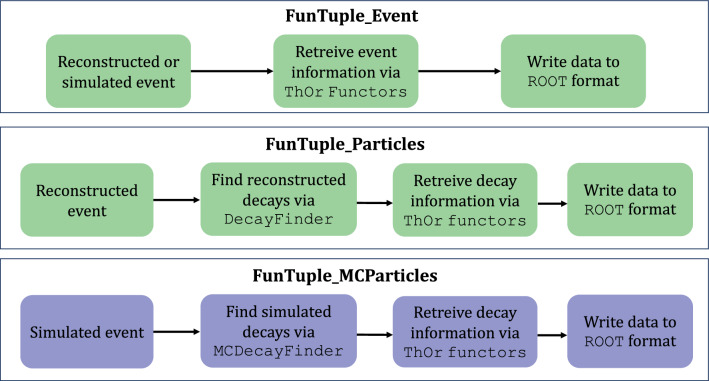
Data flow diagram of the three flavours of FunTuple component

The FunTuple_Event component processes input data comprising reconstructed or simulated events, where each event represents a single LHC bunch crossing. It acquires event-level information (for example, the number of charged particles in the event), using thread-safe ThOr functors that are specialised c++classes developed for utilisation in the second stage of the LHCb trigger system [25, 26, 35]. The component then stores this extracted information from ThOr functors in an ROOT N-tuple file. The FunTuple_Particles component functions on reconstructed events and identifies specific reconstructed decays by utilising the decay-finding tool DecayFinder [27] explained in Sect. 2.1. It further retrieves essential details regarding parent and children particles (for example magnitude of the transverse moment) through ThOr functors and records this information in an ROOT file. Similarly, the FunTuple_MCParticles component shares similarities with FunTuple_Particles, but it processes simulated events instead, and captures information about simulated decays. For an illustrative representation of the data flow encompassing these three approaches, refer to Fig. 2. Each aspect of the data-flow diagram is further elaborated in the following sections.

The instantiation of the three flavours of the FunTuple component in Python is exemplified in Listings 1, 2, 3. As depicted, the user is required to provide the name and tuple_name attributes for all three flavours. The name attribute defines the component’s name and the name of the corresponding TDirectory in the output ROOT file. On the other hand, the tuple_name attribute defines the name of the TTree in the ROOT file. The fields attribute can only be defined for FunTuple_Particles and FunTuple_MCParticles and is used to select specific decays within an event and define the corresponding TBranches in the output file. For a detailed exploration of this attribute, see Sect. "Finding Decays in an Event". The variables attribute is used to specify the observables to be computed for each event or decay. In the case of FunTuple_Event, only event-level observables can be defined. Conversely, for FunTuple_Particles and FunTuple_MCParticles, both decay-level and event-level observables can be specified. The latter is achieved by defining an optional event_variables attribute. It is worth noting that the FunTuple component automatically writes certain event information, such as the run and event numbers,^4^ to the output file by default. For a more comprehensive discussion on the variables attribute, refer to Sect. "Retrieve Event and Decay Information". Finally, the inputs attribute refers to the Transient Event Store (TES) location, indicating the data pertaining to a given event cycle that will be processed. Subsequently, the processed information is stored in the output ROOT file, which is further elaborated on in Sect. "Writing of Retrieved Information".

The FunTuple component also incorporates several essential counters to monitor the data processing. These counters include tracking the number of processed events, the count of non-empty events for each selected particle, and the tally of events with multiple candidates for each chosen particle. Upon completing the data processing, the results of these counters are displayed to the users. To ensure effective error handling, the component employs a custom error handling class that inherits from the StatusCode class implemented in Gaudi. This custom implementation enables the component to raise specific exceptions in targeted scenarios. For example, if a particular ThOr functor encounters difficulties and cannot compute an observable for a given event, the component raises an exception to promptly notify the user of the issue. Additionally, the FunTuple component takes measures to validate the input attributes both on the Python and c++sides, ensuring the correctness of the provided data. Moreover, the development process includes the creation of several tests and examples, see Sect. "Test Suite, Examples and Performance".

**Listing 1:**
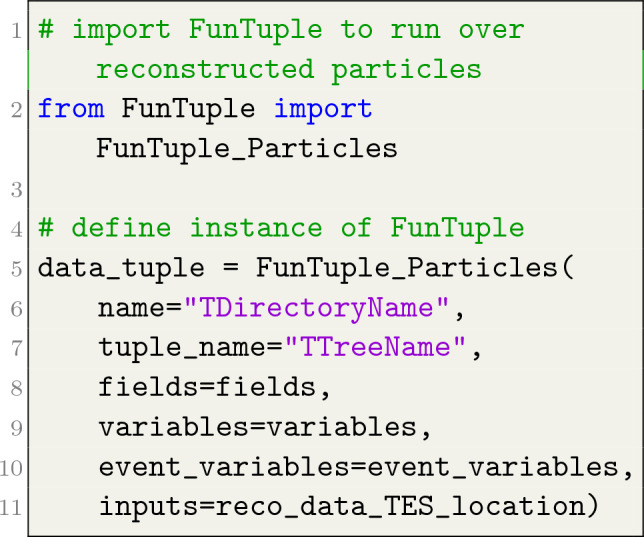
FunTuple_Particles instance

**Listing 2:**
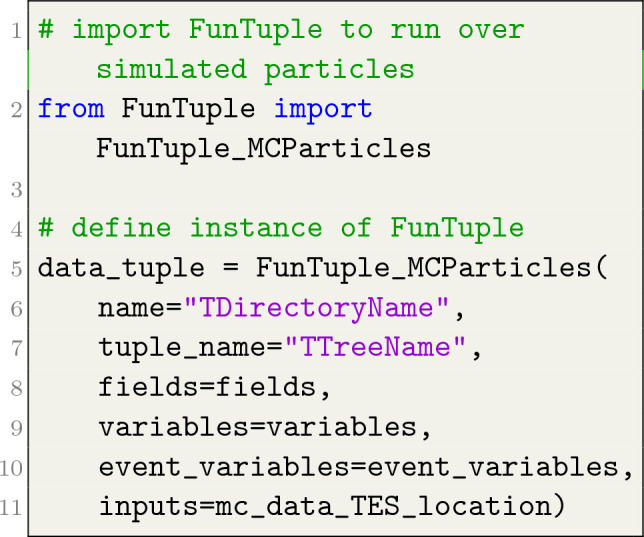
FunTuple_MCParticles instance

**Listing 3:**
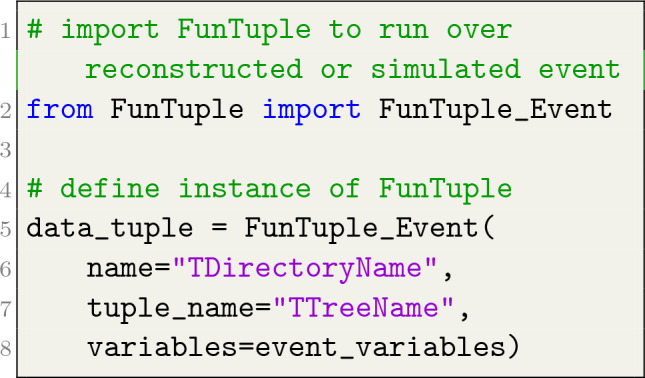
FunTuple_Event instance

### Finding Decays in an Event

Given the distinct event models for reconstructed and simulated events, the FunTuple component employs two separate Gaudi tools for decay identification. Specifically, FunTuple_Particles relies on the Gaudi tool [36] DecayFinder [37], while FunTuple_MCParticles utilises the MCDecayFinder tool [38]. Both of these tools utilise the boost library [39, 40] to parse decay descriptors. The names of particles used in the decay descriptor, along with their associated properties, are stored in the LHCb conditions database (CondDB) [41], and are retrieved through the ParticlePropertySvc [42] service.

To isolate a particular decay process within an event and select a particle within the decay chain, the user is required to provide a fields attribute to either the FunTuple_Particles or the FunTuple_MCParticles instance. The fields attribute takes the form of a string dictionary. Here, the key corresponds to the particle alias, serving as a prefix to label the TBranch in the resulting output file. On the other hand, the associated value denotes the decay descriptor employed to filter and select the particles participating in a distinct reconstructed or simulated decay process. A practical illustration of the fields attribute configuration is shown in Listing 4.

**Listing 4:**
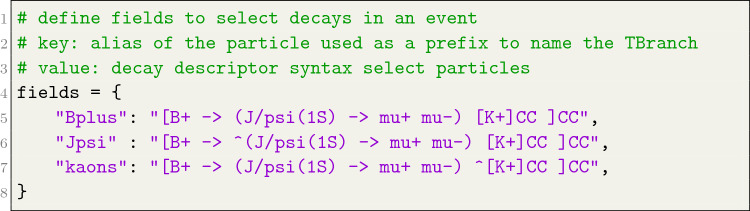
Example definition of the fields attribute

A correct syntax for the decay descriptor is crucial in the selection of the particles within a given decay process. A straightforward decay descriptor such as "B+ -> J/psi(1 S) K+" is employed to select all decays of a ${{B} ^+}$ meson into a ${J \hspace{-1.66656pt}/\hspace{-1.111pt}\psi {(1S)}}$ meson and a ${K} ^+$ meson. For the inclusion of charge-conjugate decays, users can encapsulate the decay descriptor in square brackets and append the CC keyword, such as "[B+ -> J/psi(1 S) K+]CC". This syntax covers both ${{{B} ^+}} \!\rightarrow {{J \hspace{-1.66656pt}/\hspace{-1.111pt}\psi {(1S)}}} {{K} ^+}$ and ${{{B} ^-}} \!\rightarrow {{J \hspace{-1.66656pt}/\hspace{-1.111pt}\psi {(1S)}}} {{K} ^-}$ decays. Alternatively, the []CC notation can also be used around an individual particle, e.g., "B+ -> J/psi(1 S) [K+]CC", encompassing both ${{{B} ^+}} \!\rightarrow {{J \hspace{-1.66656pt}/\hspace{-1.111pt}\psi {(1S)}}} {{K} ^+}$ and ${{{B} ^+}} \!\rightarrow {{J \hspace{-1.66656pt}/\hspace{-1.111pt}\psi {(1S)}}} {{K} ^-}$ decays.^5^ To target a specific particle within a decay, the caret symbol (${\hat{{\phantom{a}}}}$) is employed. For instance, "B+ -> J/psi(1 S) ${\hat{{\phantom{a}}}}$K+" selects the ${K} ^+$ meson, while excluding the caret symbol selects the parent particle. In cases of identical particles in the final state, the FunTuple component ensures distinct C++ objects for each identical particle instance. For example, "B+ -> ${\hat{{\phantom{a}}}}$pi+ pi- pi+" and "B+ -> pi+ pi- ${\hat{{\phantom{a}}}}$pi+" would choose two distinct instances of a ${\pi } ^+$. In the context of simulations, the FunTuple_MCParticles component utilises the LoKi decay finder [43]. This finder offers the flexibility to incorporate various arrow types within the decay descriptor syntax [43, 44]. Each arrow type allows users to selectively include simulated particles based on distinct criteria. For instance, the => arrow type signifies the inclusion of an arbitrary number of additional photons stemming from final state radiation of charged particles when matching the decay.

### Retrieve Event and Decay Information

To extract essential information related to either the event or individual particles within a decay chain, users are required to furnish the variables or event_variables attribute to FunTuple. The variables attribute functions as a python dictionary in which the key corresponds to the particle name previously defined in the fields attribute. The corresponding value is an instance of a FunctorCollection, which acts as a collection of ThOr functors, effectively resembling a dictionary itself, with the key representing the variable name and the value denoting a ThOr functor. Within the context of the FunTuple component, these ThOr functors are just-in-time (JIT) compiled and employed on the particle instance to retrieve the desired information. Notably, a key labelled ALL holds a special significance within the definition of the variables. Any FunctorCollection associated with the ALL key is applied to all particles specified in the fields attribute. In contrast, the event_variables attribute takes the form of an instance of FunctorCollection. The enclosed ThOr functors are designed to provide information at the event level. The specifics of how to define the variables and event_variables attributes are illustrated in Listing 5.

**Listing 5:**
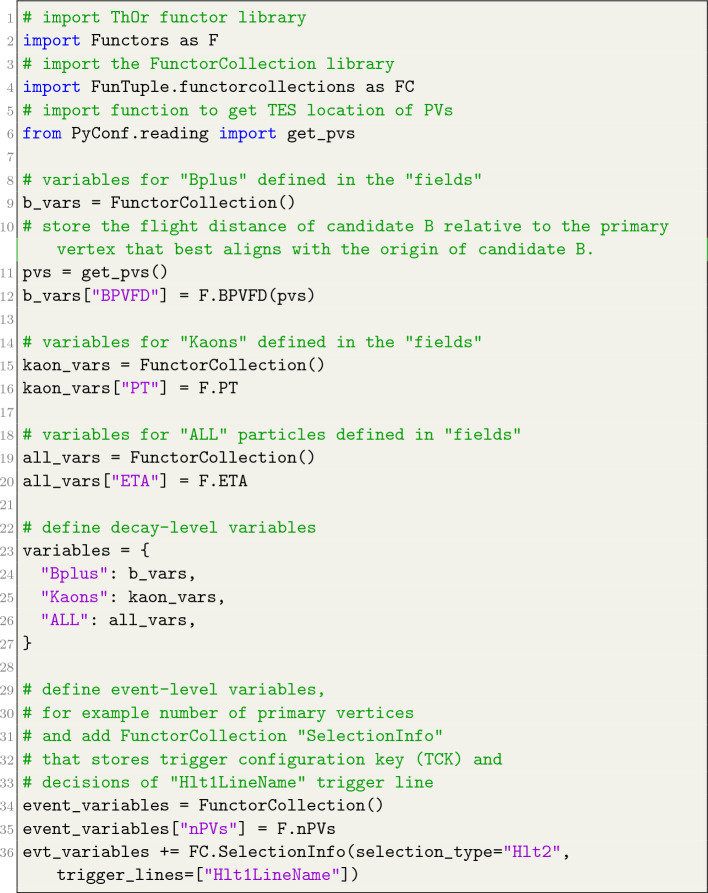
Example definition of the variables and event_variables attribute

The FunTuple component utilises the flexibility inherent in ThOr functors to extract a diverse array of information from the event. These functors are adaptable enough to accept multiple reconstructed objects as input, enabling the computation of associated information. For instance, consider the functor designed to calculate the flight distance of a particle. To achieve this, the functor takes both the reconstructed primary vertices and the reconstructed particle as input arguments. The usage of this specific functor (BPVFD) is shown in Listing  5.

The functors support all fundamental mathematical operators, including addition, subtraction, multiplication, and division. In addition, they can undergo transformations such as fmath.log(F.CHI2/F.NDOF), which, when applied to a reconstructed track, yields the track’s $\chi ^2$ per degree of freedom. Furthermore, the output from one ThOr functor can be passed as input to other ThOr functors through a mechanism known as *composition*. This proves particularly advantageous when users seek to compute an observable that relies on the outcomes of other observables. All these functionalities are harnessed to provide users with an range of observables via a pre-defined FunctorCollection instance, which is intended for use in conjunction with FunTuple. An illustrative example is the SelectionInfo collection, which gathers the functors employed to store the trigger configuration key (TCK) and the event’s trigger line decision. Listing 6 outlines the definition of this collection, with its application showcased in Listing 5.

In this listing, the SelectionInfo collection is designed to take two main inputs: the type of selection, which can be any of the three stages (Hlt1, Hlt2, or Sprucing), and a list of trigger or Sprucing lines. In response, it generates a FunctorCollection that incorporates two functors: F.TCK for storing TCK information and F.DECISION for storing the trigger decision of the specified selection line. Such collections do not expose the users to the technical intricacies involved in retrieving the requested information. In this particular case, the involved functors require the DecReport object, which is obtained from the DaVinci framework via the get_decreports function. Furthermore, users maintain the flexibility to add, merge or remove observables within these collections, enabling them to create their customised collections. Multiple collections have been developed and continue to be actively expanded, accompanied by relevant unit tests within the DaVinci framework.

**Listing 6:**
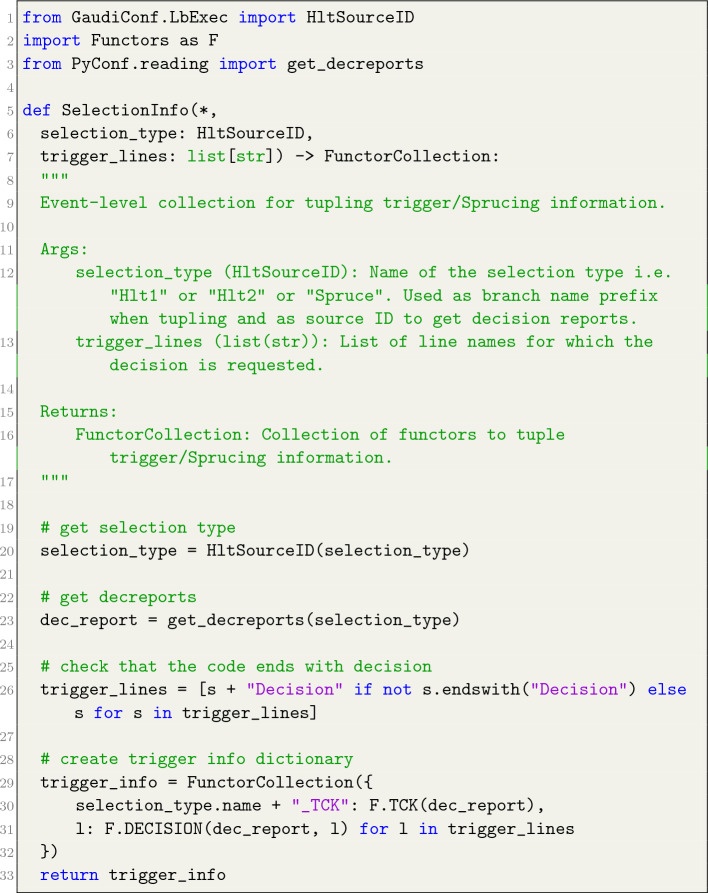
Example definition of SelectionInfo collection

### Writing of Retrieved Information

The ThOr functors, utilised for retrieving reconstructed or truth-level information, are capable of encapsulating data in a diverse range of formats. These functors can not only return basic c++types, but also yield complex objects pertaining to the LHCb software framework. Subsequently, the extracted information is recorded within the ROOT file, where each TBranch corresponds to a scalar or an array of basic c++types. FunTuple accommodates diverse data object types returned by ThOr functors. An illustrative example is the functor F.STATE, which retrieves the complete state of a reconstructed track, i.e. instance of LHCb::State, which includes information on track position, charge, momentum, track slopes, and the associated covariance matrix. FunTuple processes this returned class instance, enabling the writing of multiple observables into the ROOT file from a single functor. In this context, FunTuple supports various variable types, and the list is rapidly expanding. These include three vectors, four vectors, SIMD versions of arrays, matrices of both symmetric and non-symmetric nature with arbitrary dimensions, containers spanning arbitrary dimensions, various enumerations, e.g. vertex type, track state, as well as $\texttt {std::optional}<\texttt {T}>$ constructs and $\texttt {std::map}<\texttt {std::string, T}>$ structures, where T represents any of the supported types. Additionally, extending support for other custom classes is remarkably straightforward.

As of the preparation of this document, the FunTuple component utilises the GaudiTupleAlg tool [42], which registers an entry in the ROOT file in a thread-safe manner. However, this tool does not provide full support for various complex data objects returned by ThOr functors; such support is exclusively offered by FunTuple. The transition to ROOT’s RNTuple is planned for the future with subsequent retirement of the GaudiTupleAlg tool.

### Test Suite, Examples and Performance

FunTuple includes an extensive set of examples and tutorials for users, along with a dedicated test suite based on pytest [45]. Both unit tests and “physics tests" are crafted to assess various functionalities of the component, ensuring its reliability. Additionally, an application test accompanies each example job run in continuous integration, serving to guarantee correct functionality consistently.

Comprising just over 100 unit tests and some 40 “physics tests", the test suite currently in place evaluates various aspects of the FunTuple behaviour. These include checking for appropriate error messages in case of incorrect configurations, ensuring correct output with specified settings, validating expected numbers written to the ROOT file, testing the behaviour of FunctorCollections, assessing the output of FunTuple when run with different event models, and more. The test coverage for both FunTuple and the decay finder stands at an impressive 100%.

While a comprehensive performance analysis of FunTuple is not the focus of this paper, a brief overview is provided. In offline analysis, computing hundreds of observables is common. Recording 740 observables using ThOr functors for 1000 events takes 3 min, with JIT compilation of about 200 functors taking 84 s. Post-compilation, a functor cache is created, reducing overhead in both online and offline data processing. The Python front-end of FunTuple assists in early error detection in configurations, and the performance impact from combining C++/Python is minimal relative to functor execution time.

## Interface with Other Gaudi Algorithms

In the LHCb framework, the execution of multiple algorithms within the offline data processing pipeline is a common necessity. Notable examples of such algorithms encompass the DecayTreeFitter[46], which fits complete decay chains with optional primary vertex constraints or mass constraints on intermediary states; the MCTruthAndBkgCatAlg algorithm [27], which is used to extract truth-level information from reconstructed objects in simulations; the ParticleCombiner algorithm [27], for combining basic particles into composite entities; among others. These algorithms can be employed in conjunction with the FunTuple component to process and store data. A practical illustration of FunTuple in synergy with DecayTreeFitter and MCTruthAndBkgCat is presented in Listing 7.

In this listing, the DecayTreeFitter and MCTruthAndBkgCat algorithms operate on reconstructed ${{{B} ^+}} \!\rightarrow {{J \hspace{-1.66656pt}/\hspace{-1.111pt}\psi {(1S)}}} {{K} ^+}$ decays. Under the hood, both algorithms construct a relation table linking the reconstructed object with a related object that holds pertinent information. For MCTruthAndBkgCat, the related object is the associated simulation object, harbouring truth-level information; conversely, for DecayTreeFitter, the related object corresponds to the output of the decay tree fitting process. To extract the relevant information, the reconstructed object is mapped to the related object, and the ThOr functor is applied to the related object. This entire process is executed within the __call__ method of both the MCTruthAndBkgCat and DecayTreeFitter algorithms. For example, in Listing 7, calling MCTRUTH(F.FOURMOMENTUM) establishes a mapping between the reconstructed ${{{B} ^+}} \!\rightarrow {{J \hspace{-1.66656pt}/\hspace{-1.111pt}\psi {(1S)}}} {{K} ^+}$ decay and the corresponding simulation object. Subsequently, the F.FOURMOMENTUM functor is employed on the simulation object to retrieve the true four-momentum of the B+ meson. A similar approach is followed for the DTF(F.FOURMOMENTUM), with the distinction that the four-momentum of the ${{B} ^+}$ meson is stored following the decay tree fit, incorporating mass constraint on the ${J \hspace{-1.66656pt}/\hspace{-1.111pt}\psi {(1S)}}$ meson and primary vertex constraint.

The interaction between FunTuple and other Gaudi algorithms is fortified by a fail-safe mechanism. When either of the algorithms encounters failure, such as the absence of corresponding truth-level information or unsuccessful decay tree fitting, the ThOr functors and FunTuple are equipped to handle the situation. If the ThOr functor returns data of floating-point type, the FunTuple component automatically records Not a Number (NaN) in the ROOT file. Conversely, if the ThOr functor returns an integral type, the invalid value needs to be explicitly defined using the F.VALUE_OR functor, exemplified in Listing 7.

**Listing 7:**
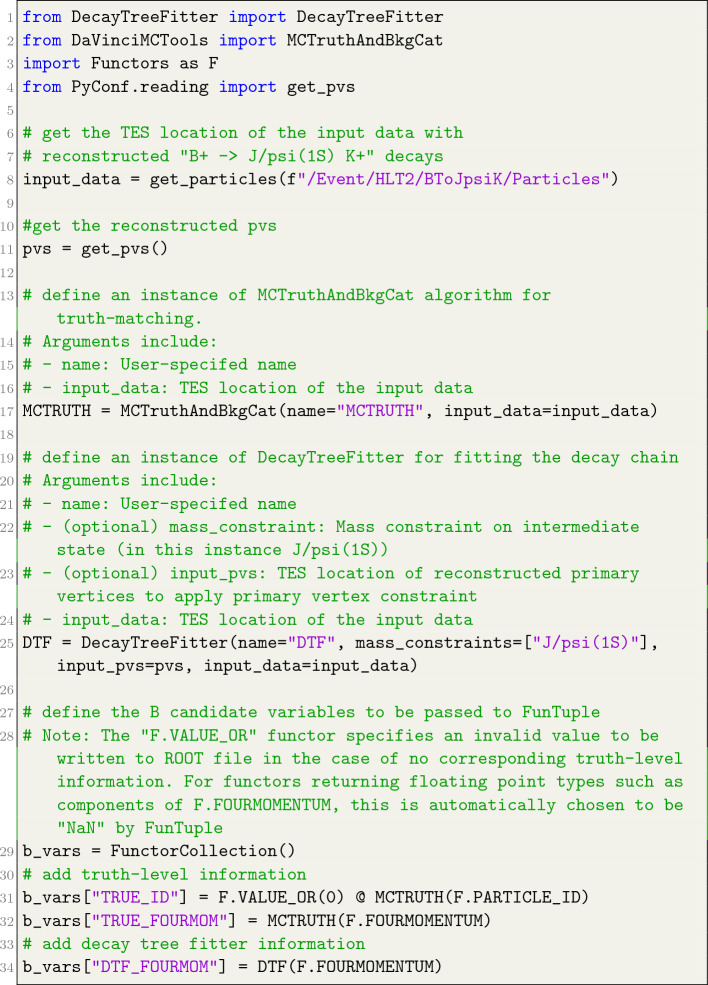
Usage of the truth-matching (MCTruthAndBkgCat) and decay tree fitting (DecayTreeFitter) algorithms in conjuntion with FunTuple. Note that the FunTuple definition shown in Listing 1 does not change

## Summary and Conclusions

**Fig. 3:**
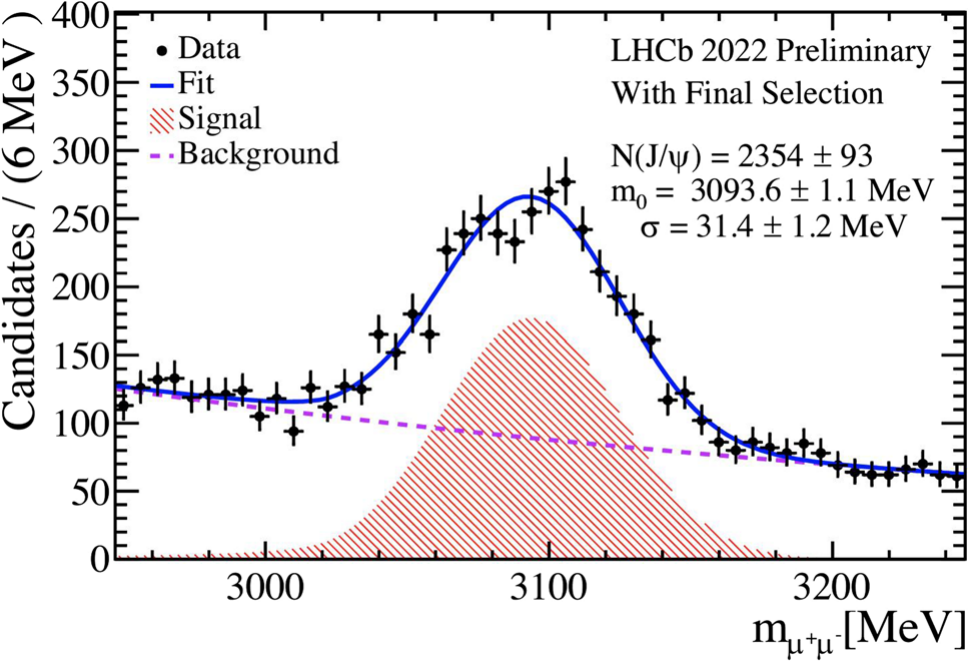
Invariant mass of the ($\mu ^-$ $\mu ^+$) system showing the ${J \hspace{-1.66656pt}/\hspace{-1.111pt}\psi {(1S)}}$ peak for LHCb data collected during the current Run 3 commissioning data taking period in 2022 [47]

This paper introduces the FunTuple component, designed to support offline data processing for the LHCb experiment during the current Run 3 and subsequent runs. Its primary purpose is to facilitate the storage of experiment-acquired data in the ROOT format, optimising it for subsequent offline analysis. Currently, the component plays a vital role in various early measurement analyses of LHCb data collected during the current Run 3 data taking period. An example of the processed data using FunTuple is displayed in Fig. 3, showcasing the reconstructed mass of the ${{J \hspace{-1.66656pt}/\hspace{-1.111pt}\psi {(1S)}}} \!\rightarrow {\mu ^-} {\mu ^+}$ decay from LHCb data gathered in 2022 during commissioning [47]. The figure shows the signal ${{J \hspace{-1.66656pt}/\hspace{-1.111pt}\psi {(1S)}}} \!\rightarrow {\mu ^-} {\mu ^+}$ component in red-filled histogram and the background component in dotted purple line. The background component involves random combinations of muons from different part of the event. The total fit component, composed of both signal and background, is shown in solid blue line and the data points are shown in black dots. The number of signal candidates is estimated to be $N_{{{J \hspace{-1.66656pt}/\hspace{-1.111pt}\psi {(1S)}}}} = 2354 \pm 93$ with mass $m_0 = 3093.6 \pm 0.2$ $\textrm{MeV}/c^{2}$and width $\sigma = 9.1 \pm 0.2$ $\textrm{MeV}/c^{2}$to be consistent with the known ${J \hspace{-1.66656pt}/\hspace{-1.111pt}\psi {(1S)}}$ mass and width [48].

Furthermore, the FunTuple component is built upon the Gaudi functional framework, and it offers a user-friendly Python interface. Its templated design enables it to accommodate various types of input data, including reconstructed and simulated events, and it supports the processing of both event-level and decay-level information. Additionally, this templated design allows the component to support new event models, based on *SoA* data structure in the future, facilitating vectorised processing of data. Of particular importance is its ability to ensure equivalence between trigger-computed observables and those subjected to offline analysis. This achievement is made possible through the integration of the ThOr functors, adept at computing topological and kinematic observables. Users also have substantial flexibility, enabling them to personalise the range of observables stored within the ROOT file. The component is also thoroughly validated through a series of unit-tests and pytest tests to ensure its reliability. In conclusion, the unique attributes of the FunTuple component establish it as a robust tool for offline data processing at the LHCb experiment making it essential for Run 3 and beyond.

## Data Availability

The software component developed here is open source and is available in the gitlab via the link: https://gitlab.cern.ch/lhcb/DaVinci. The tests and examples use LHCb Run 3 data, which is not accessible openly.

## Footnotes

1 The data collection from Run 3 of LHC is currently ongoing; however, the core developments emphasised in this paper transpired prior to its commencement.

2 There were also alternative Python-based algorithms like Bender [18, 19] for Run 1/2 data processing.

3 There are plans in the future to write ROOT RNTuple, which has been designed to address performance bottlenecks and shortcomings of ROOT current state of the art TTree [28].

4 Both run and event numbers are used to uniquely identify an event in the LHC experiments.

5 The charge-violating decays are often reconstructed at LHCb to serve as proxies for the study of sources of background.
